# Multimodality Imaging Reveals Rare Hereditary Transthyretin Amyloidosis Mimicking Hypertrophic Cardiomyopathy

**DOI:** 10.1016/j.jaccas.2026.108961

**Published:** 2026-09-23

**Authors:** Maria Panelo, Yoel Tuya-Acosta, Adriana Soto-Priore, E. Javier Alderete-Parisi, Diana García-del-Barco Herrera, Alicia Maceira

**Affiliations:** a Cardiovascular Imaging Unit, Ascires Biomedical Group, Barcelona, Spain; b Cardiology Department, Barcelona Hospital, Barcelona, Spain; c Cardiology Department, Martorell Sant Joan de Déu Hospital Foundation, Barcelona, Spain; d Cardiology Department, Hospital Universitari de Bellvitge, Barcelona, Spain; e Neuroprotection Project, Department of Pharmaceuticals, Biomedical Research Division, Center for Genetic Engineering and Biotechnology (CIBG), Havana, Cuba; f Latin American School of Medicine, Havana, Cuba; g Biotechnology Joint Innovation Center Yongzhou Zhon Gu Biotechnology Co. LTD. Yongzhou Economic and Technological Development Zone, Hunan Province, China; h Cardiovascular Imaging Unit, Ascires Biomedical Group, Valencia, Spain; i Department of Medicine, Faculty of Health Sciences, UCH-CEU University, Valencia, Spain

**Keywords:** cardiac magnetic resonance, cardiomyopathy, diastolic heart failure, genotype, imaging, nuclear medicine

## Abstract

**Background:**

Distinguishing hypertrophic cardiomyopathy from phenocopies remains a diagnostic challenge in younger patients presenting with unexplained ventricular hypertrophy.

**Case Summary:**

A 52-year-old Moroccan woman previously diagnosed with nonobstructive hypertrophic cardiomyopathy presented with recurrent pericardial chest pain, poorly tolerating angiotensin-converting enzyme inhibitors and beta-blockers. Cardiac magnetic resonance showed asymmetric hypertrophy and papillary muscle hypertrophy suggestive of hypertrophic cardiomyopathy, with markedly elevated native T1 mapping and extracellular volume. Bone scintigraphy subsequently demonstrated intense cardiac tracer uptake, consistent with transthyretin cardiac amyloidosis. Genetic testing confirmed a rare pathogenic *TTR* variant, p.Lys35Asn. Neurologic evaluation revealed moderate to severe sensorimotor polyneuropathy. Disease-modifying therapy with vutrisiran was initiated within a multidisciplinary care program.

**Discussion:**

Current cardiomyopathy guidelines emphasize the importance of recognizing systemic red flags in hypertrophic phenotypes. Multimodality imaging is critical to establish diagnosis of transthyretin cardiac amyloidosis and allow timely initiation of disease-modifying therapy.

**Take-Home Message:**

Unexplained hypertrophy with systemic manifestations should prompt evaluation for transthyretin amyloidosis.

## Visual Summary

**Figure:**
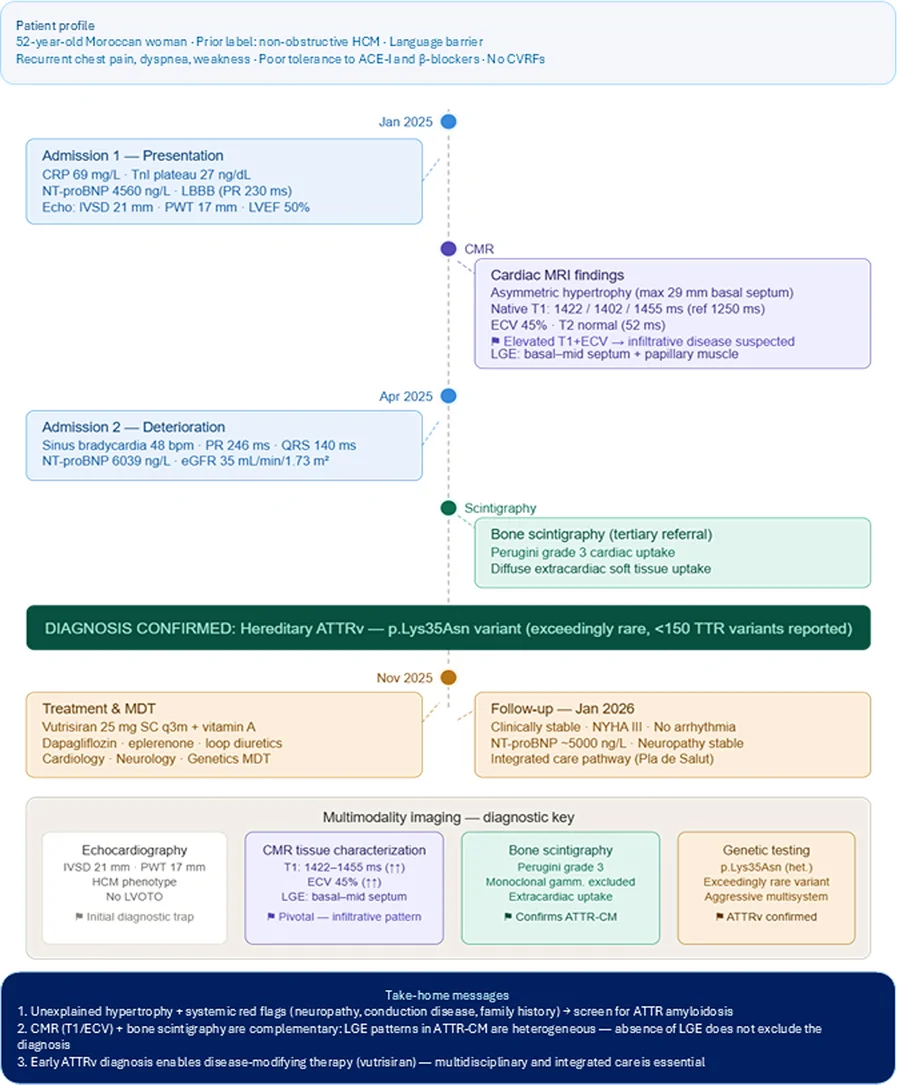

**Visual Summary:**
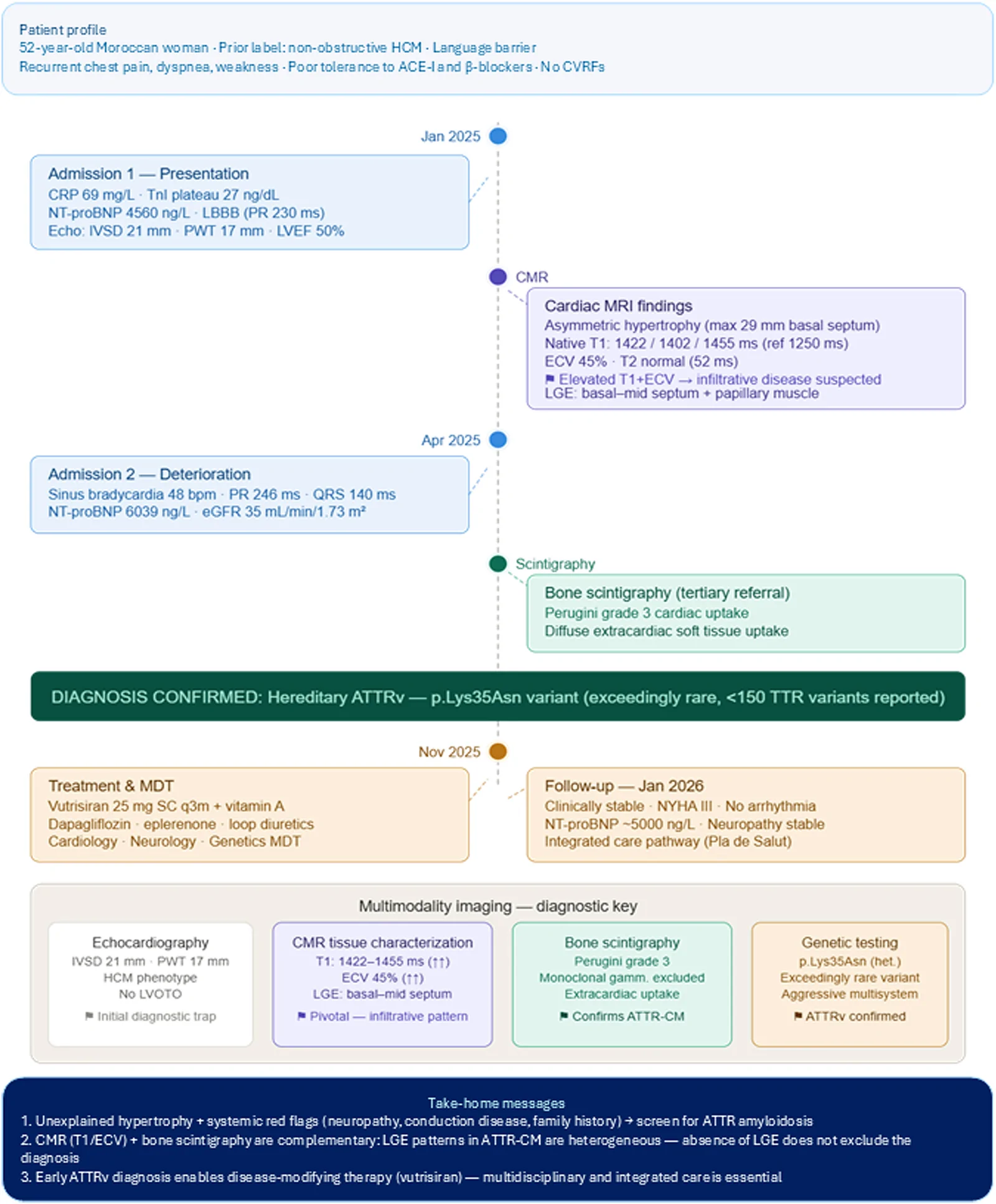
Diagnostic Timeline and Multimodality Imaging Findings in a 52-Year-Old Woman With Hereditary Transthyretin Amyloidosis (ATTRv, p.Lys35Asn) Initially Misdiagnosed as Nonobstructive Hypertrophic Cardiomyopathy The top panels summarize the patient profile. The central timeline depicts the chronological sequence of clinical events from January 2025 to January 2026, including 2 hospital admissions, CMR, bone scintigraphy, genetic confirmation, and initiation of disease-modifying therapy. The imaging comparison panel highlights the complementary diagnostic roles of echocardiography (initial phenotypic trap), CMR tissue characterization (markedly elevated native T1 mapping and extracellular volume, consistent with infiltrative disease), bone scintigraphy (Perugini grade 3, confirming transthyretin cardiac amyloidosis), and genetic testing (rare pathogenic variant, p.Lys35Asn). The lower panel summarizes the 3 principal take-home messages. ACE-I = angiotensin-converting enzyme inhibitor; ATTR-CM = transthyretin cardiac amyloidosis; ATTRv = hereditary transthyretin amyloidosis; CMR = cardiac magnetic resonance; CRP = C-reactive protein; CVRF = cardiovascular risk factor; ECV = extracellular volume; HCM = hypertrophic cardiomyopathy; IVSD = interventricular septal thickness in diastole; LBBB = left bundle branch block; LGE = late gadolinium enhancement; LVEF = left ventricular ejection fraction; LVOTO = left ventricular outflow tract obstruction; MDT = multidisciplinary team; NT-proBNP = N-terminal pro–B-type natriuretic peptide; PWT = posterior wall thickness; SC = subcutaneous; Tn1 = troponin I; TTR = transthyretin.

## History of Presentation

A 52-year-old Moroccan woman with a prior diagnosis of nonobstructive hypertrophic cardiomyopathy was admitted in January 2025 with acute pleuritic chest pain and an inflammatory syndrome.

Communication was limited because of a complete language barrier. She reported that in her home country she had been told she had an “enlarged heart.” There were no conventional cardiovascular risk factors.

On admission, inflammatory markers were elevated, with C-reactive protein of 69 mg/L. Cardiac biomarkers showed a plateau pattern with troponin I values of 27-26-26 ng/dL and N-terminal pro–B-type natriuretic peptide (NT-proBNP) of 4,560 ng/L. Autoimmune testing revealed positive rheumatoid factor, ANA 1:80 (homogeneous), elevated U1-RNP and Ro60 antibodies, with normal complement and negative antiphospholipid antibodies.

Electrocardiography demonstrated sinus rhythm at 66 beats/min, first-degree atrioventricular block with PR interval 230 ms, QRS 130 ms, and left bundle branch block (LBBB) with secondary repolarization abnormalities.

**Figure 1:**
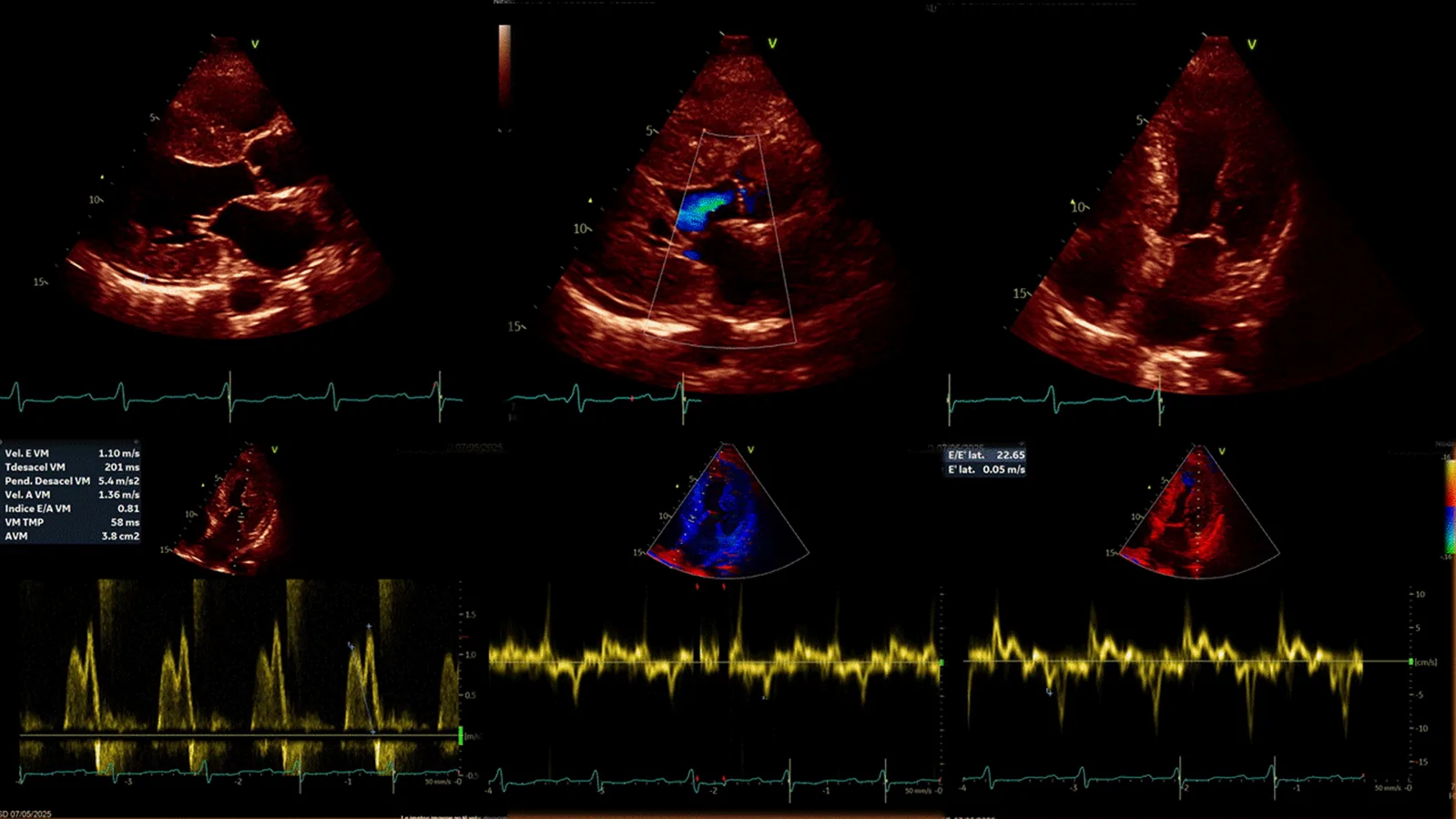
Echocardiography

Transthoracic echocardiography revealed a nonobstructive hypertrophic cardiomyopathy phenotype (interventricular septum thickness in diastole: 21 mm, posterior wall thickness: 17 mm) with borderline left ventricular systolic function (ejection fraction: 50%) and minimal posterior pericardial effusion (Figure 1).

Despite treatment with nonsteroidal anti-inflammatory therapy, angiotensin-converting enzyme inhibitors, and low-dose beta-blocker therapy, the patient experienced persistent symptoms with repeated emergency consultations for chest pain, dyspnea, and weakness.

In April 2025 she was readmitted with dizziness, lower limb weakness, and nocturnal palpitations associated with dyspnea. Blood pressure was 106/55 mm Hg. Electrocardiography showed sinus bradycardia at 48 beats/min with PR interval 246 ms and LBBB slightly more aberrant (QRS 140 ms). NT-proBNP increased to 6,039 ng/L, and renal function deteriorated transiently (estimated glomerular filtration rate: 35 mL/min/1.73 m^2^).

## Past Medical History

The patient had been diagnosed with nonobstructive hypertrophic cardiomyopathy several years earlier. She had no known hypertension, diabetes, or dyslipidemia, nor any other relevant medical diagnosis. Her family history was notable for 2 sisters and 1 brother who died around the age of 50 years with unexplained gastrointestinal symptoms.

No prior neurological diagnosis had been documented before the current evaluation, although neurological abnormalities may have gone unnoticed during emergency department visits and cardiology assessments.

## Differential Diagnosis

The initial differential diagnosis included sarcomeric hypertrophic cardiomyopathy, inflammatory cardiomyopathy associated with pericarditis, infiltrative cardiomyopathy including amyloidosis, and, less likely, storage diseases such as Anderson-Fabry disease.

The coexistence of conduction disease, progressive symptoms, and family history raised suspicion of a systemic disorder.

The sluggish evolution with multiple visits to the emergency department and poor tolerance to angiotensin-converting enzyme inhibitors and beta-blockers could also point to cardiac amyloidosis.

Monoclonal gammopathy was excluded by serum and urine protein electrophoresis, which demonstrated low albumin and elevated α1-globulins plus γ-globulins, consistent with a reactive polyclonal hypergammaglobulinemia pattern rather than a discrete monoclonal spike. Serum and urine immunofixation were negative for any monoclonal component, effectively ruling out light chain cardiac amyloidosis, multiple myeloma, and other plasma cell dyscrasias. Serum free light chain analysis revealed a symmetrical elevation of kappa and lambda chains with a κ/λ ratio of 1.28, within the normal range.

## Investigations

**Figure 2:**
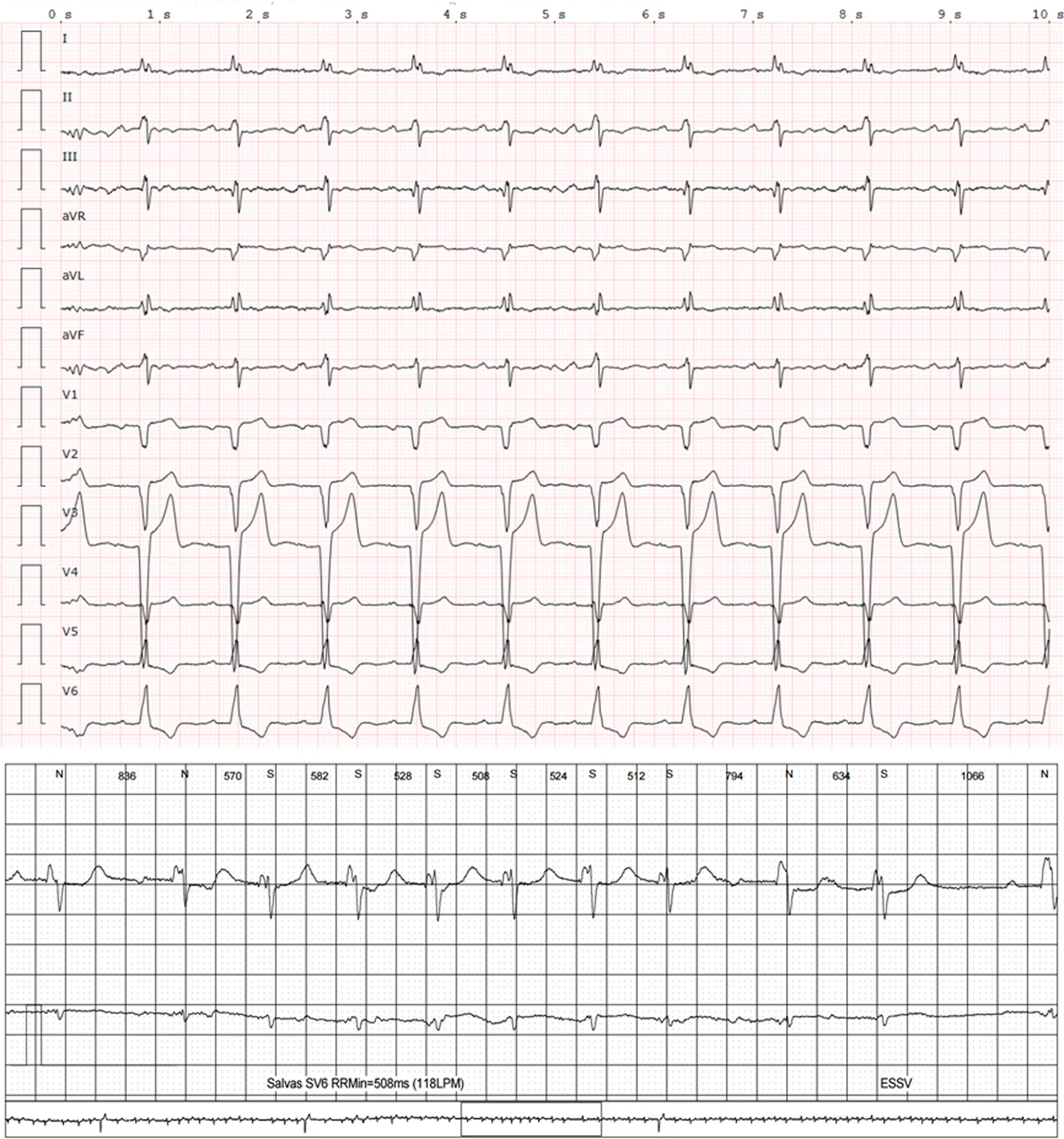
ECG and Holter Monitoring ECG = electrocardiogram.

Holter monitoring demonstrated sinus rhythm with bradycardic tendency and first-degree atrioventricular block with PR intervals up to 300 ms but no higher-grade conduction disturbances (Figure 2).

**Figure 3:**
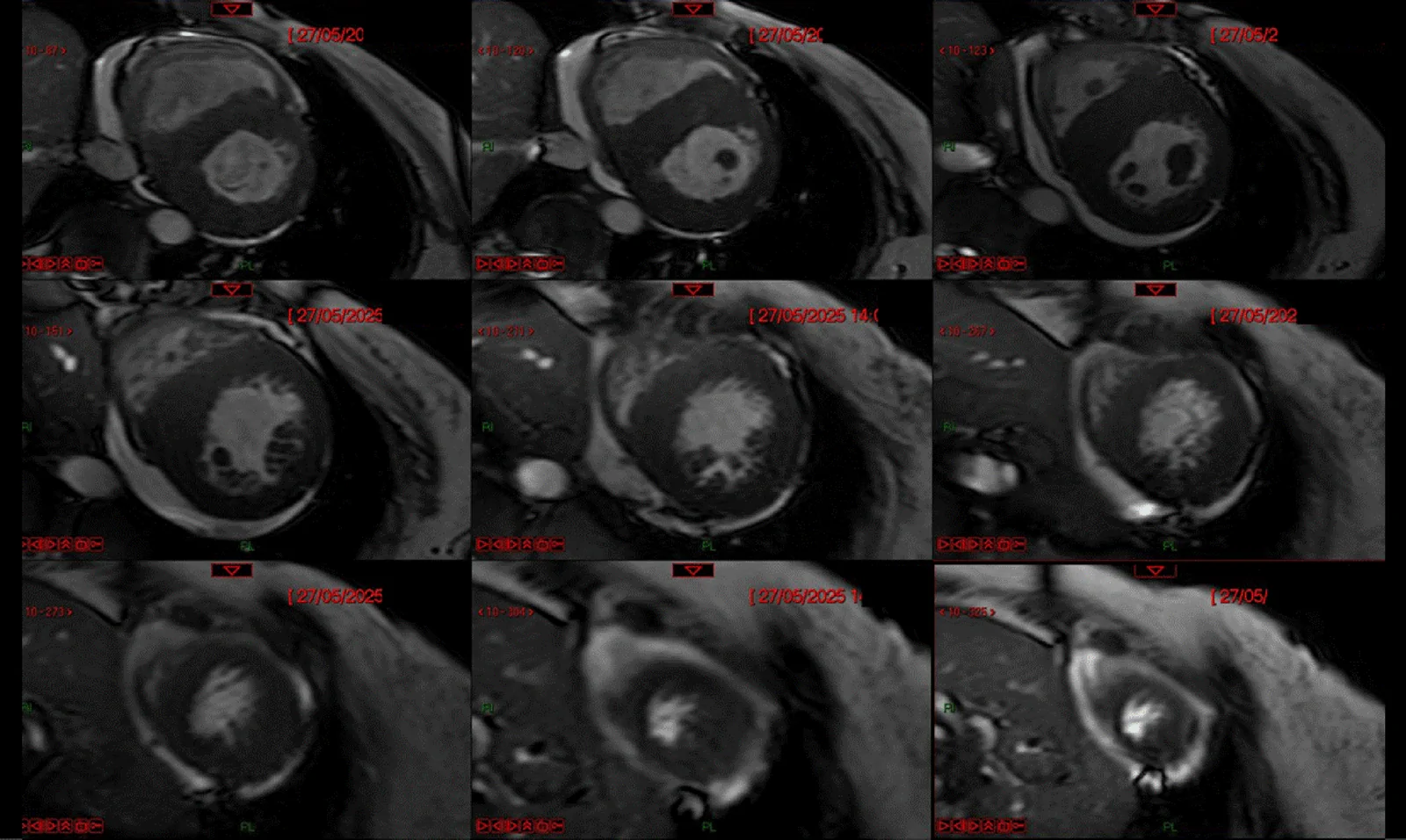
CMR Short-Axis Cine “Diagnostic Trap” CMR = cardiac magnetic resonance.

Cardiac magnetic resonance (CMR) was subsequently performed and showed circumferential pericardial effusion, maximal posteriorly, measuring up to 22 mm in the atrioventricular groove. The left ventricle was nondilated with mildly reduced systolic function (left ventricular ejection fraction: 49%), diffuse septal hypokinesia, and mild dyssynchrony related to LBBB. Myocardial mass was markedly increased with asymmetric hypertrophy following a septal helical distribution. Maximal wall thickness reached 29 mm at the basal inferoseptal junction. Papillary muscles appeared fragmented with apical insertion and hypertrophy of the anterolateral papillary muscle. Basal diaphragmatic right ventricular hypertrophy was also noted (9.5 mm) (Figure 3).

**Figure 4:**
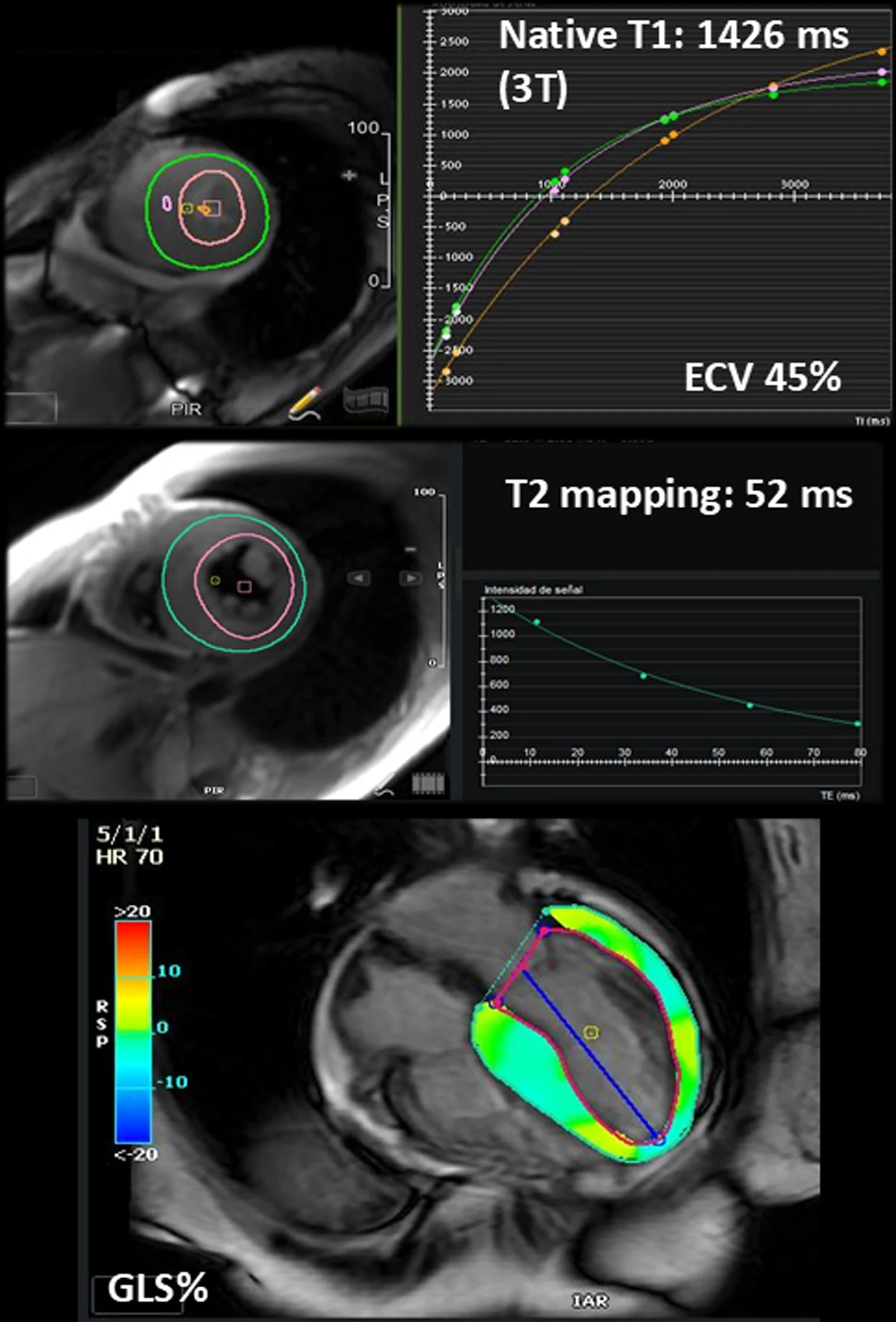
Tissue Characterization and Cardiac Magnetic Resonance Global Longitudinal Strain Percentage

Tissue characterization by CMR revealed markedly elevated native T1 mapping values: basal, 1,422 ms; mid, 1,402 ms; and apical, 1,455 ms (reference: 1,250 ms at 3-T) with an estimated extracellular volume (ECV) of 45%. It is worth noting that the mapping progression did not follow an apex-to-basal pattern, remaining dramatically elevated at all levels. Also, CMR global longitudinal strain was diffusely altered throughout the left ventricle, with no preserved apical values observed. T2 mapping was normal (52 ms) (Figure 4).

**Figure 5:**
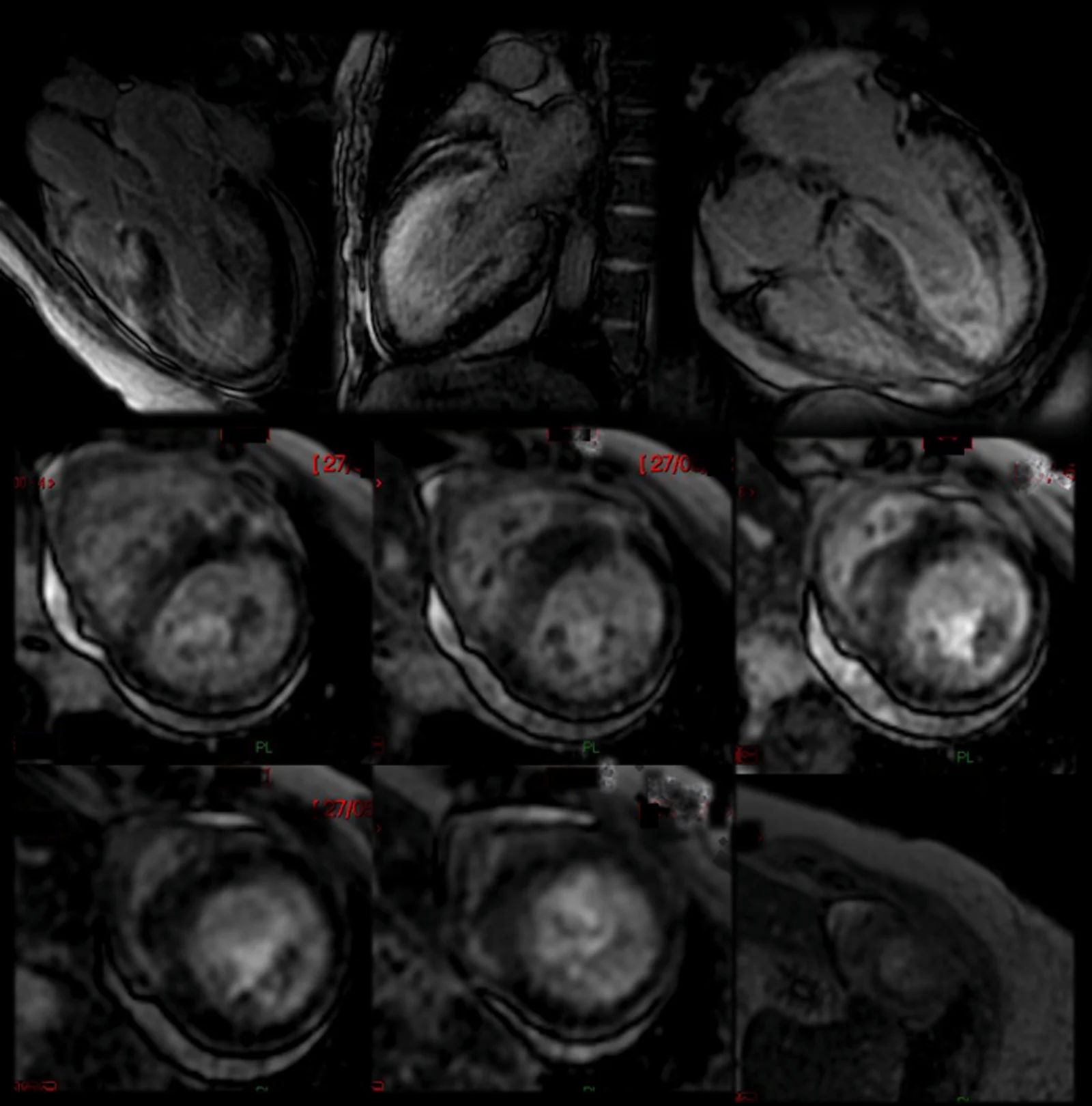
Late Gadolinium Enhancement

Late gadolinium enhancement (LGE) suggested intramyocardial fibrosis predominantly involving basal and mid septal segments and corresponding to the areas of greatest hypertrophy, with additional enhancement of the posterolateral papillary muscle (Figure 5).

Although the initial interpretation favored hypertrophic cardiomyopathy with extensive fibrosis, the markedly elevated T1 mapping values and ECV raised suspicion of infiltrative disease.

**Figure 6:**
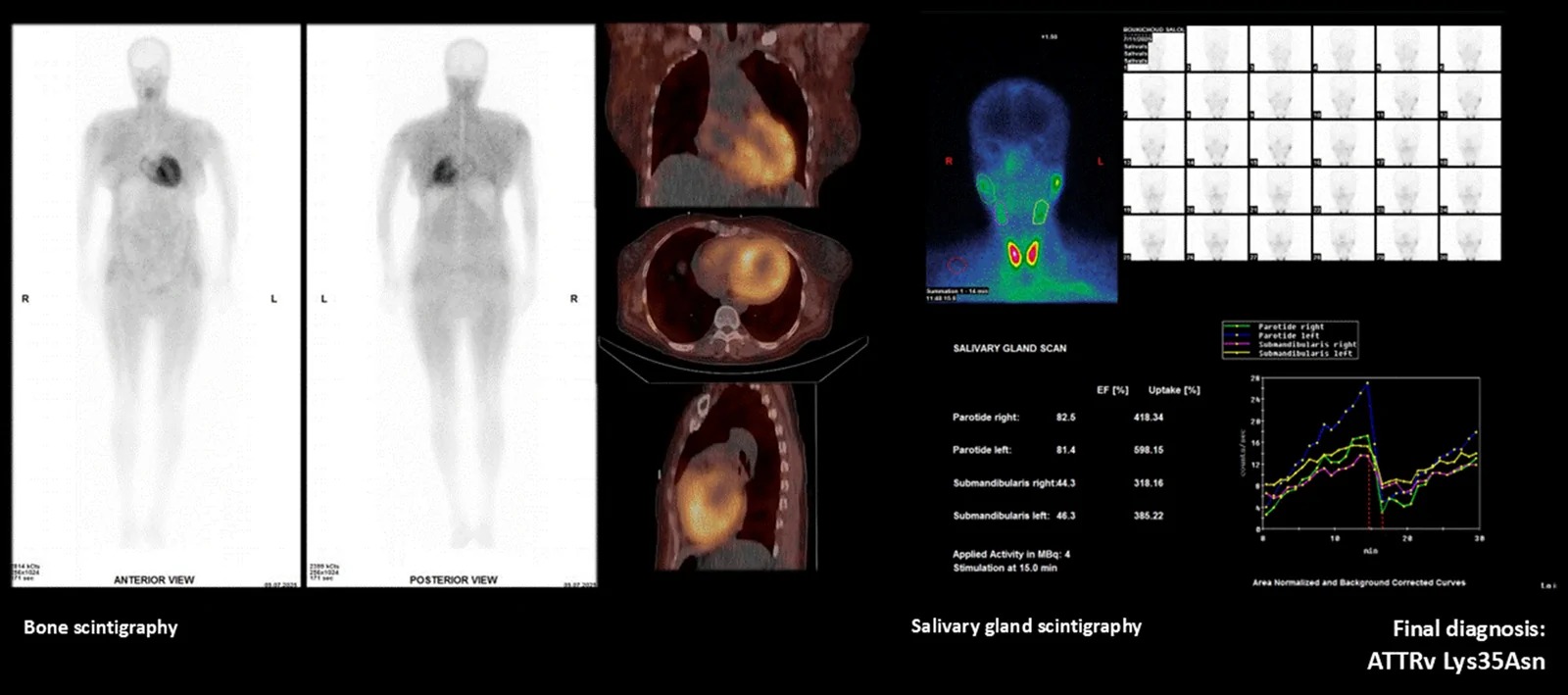
Final Diagnosis: Bone and Salivary Gland Scintigraphy

The patient was subsequently referred to a tertiary center, where bone scintigraphy demonstrated intense cardiac tracer uptake (Perugini score: 3) together with diffuse extracardiac soft tissue uptake, confirming systemic transthyretin cardiac amyloidosis (ATTR-CM) (Figure 6).

## Management

Genetic analysis identified a heterozygous pathogenic variant in the transthyretin gene, p.Lys35Asn, confirming hereditary ATTR-CM.

Neurological evaluation revealed significant peripheral involvement. Electromyography and clinical assessment demonstrated a Neuropathy Impairment Score of 65.5, consistent with moderate to severe sensorimotor neuropathy. Functional evaluation showed Polyneuropathy Disability Score II-IIIa and familial amyloid polyneuropathy stage II.

Salivary gland scintigraphy showed functional impairment of both parotid and submandibular glands, supporting multisystem disease involvement.

The case was discussed in a multidisciplinary team involving cardiology, neurology, and genetics specialists. Disease-modifying therapy with the transthyretin silencer vutrisiran was initiated, along with standard heart failure therapy including dapagliflozin, eplerenone, and loop diuretics.

## Outcome and Follow-Up

The first dose of vutrisiran was administered in November 2025. At follow-up in January 2026, the patient remained clinically stable. She reported exertional dyspnea corresponding to NYHA functional class III but no syncope or sustained arrhythmias. NT-proBNP remained elevated at approximately 5,000 ng/L. Neuropathy severity remained stable. Electrocardiography showed sinus rhythm at 64 beats/min with persistent conduction abnormalities including first-degree atrioventricular block and LBBB.

A coordinated follow-up strategy was implemented involving a local heart failure unit and a tertiary familial cardiomyopathy program. Multimodal reassessment was scheduled every 6 months.

Physical rehabilitation improved functional conditioning, and the patient maintained independence in daily activities without further hospital admissions.

## Discussion

This case illustrates the diagnostic complexity of unexplained ventricular hypertrophy and highlights the phenotypic overlap between sarcomeric hypertrophic cardiomyopathy and cardiac amyloidosis.^1^^,^^2^

ATTR-CM results from extracellular deposition of misfolded transthyretin fibrils leading to progressive myocardial infiltration, diastolic dysfunction, and conduction abnormalities. Although classically associated with increased wall thickness without marked asymmetry, substantial morphological variability has been described.^3^^,^^4^

In the present case, CMR revealed asymmetric hypertrophy and papillary abnormalities with LGE predominating in the areas of greatest hypertrophy including papillary muscles, a pattern that could suggest hypertrophic cardiomyopathy with associated fibrosis. However, markedly elevated native T1 mapping values and ECV were highly suggestive of infiltrative cardiomyopathy. While diffuse subendocardial or transmural LGE is frequently described, the LGE localization from base to mid septum can also be consistent with cardiac amyloidosis.

Current cardiomyopathy guidelines emphasize the importance of recognizing red flags suggesting systemic disease in patients with hypertrophic phenotypes. These include conduction abnormalities, peripheral neuropathy, unexplained heart failure symptoms, and suggestive family history. Additionally, given the overlap in imaging findings of hypertrophic cardiomyopathy and cardiac amyloidosis, and the availability of treatment for cardiac amyloidosis, screening for cardiac amyloidosis should likely be more widely considered in this phenotype. Although ATTR-CM most commonly affects older patients or those with a concerning family history, light chain cardiac amyloidosis (AL-CM) can occur at any age.

Bone scintigraphy with bone-seeking tracers remains a cornerstone of noninvasive diagnosis of ATTR-CM when monoclonal gammopathy has been excluded. In this patient, Perugini grade 3 cardiac tracer uptake confirmed the diagnosis.

Genetic testing identified the p.Lys35Asn heterozygous variant in the transthyretin gene. The literature describes more than 150 pathogenic variants of the *TTR* gene.^5^ However, the specific p.Lys35Asn variant remains exceedingly rare.^6^ This variant has been associated with aggressive multisystem disease, including severe neuropathy.

Vutrisiran, an RNA interference therapy reducing hepatic transthyretin production, received Food and Drug Administration approval in 2022 for hereditary ATTR with polyneuropathy and in 2025 for ATTR cardiomyopathy, and is administered subcutaneously at 25 mg every 3 months with vitamin A supplementation.^7^^,^^8^ In the HELIOS-B trial, vutrisiran vs placebo in 655 patients with ATTR cardiomyopathy demonstrated a 35% reduction in all-cause mortality in the overall population and 34% in the monotherapy cohort, along with a significant reduction in recurrent cardiovascular events, preserved functional capacity, and improved health status and quality of life.^7^, ^8^, ^9^, ^10^

Equally important in this case was the implementation of an integrated, patient-centered care model (Catalan framework: Pla de Salut de Catalunya) coordinating primary care, regional hospital resources, and tertiary specialist input, aimed at reducing fragmentation, preventing unnecessary hospitalizations, and ensuring management at the most appropriate level across the care continuum. Such coordinated strategies are particularly valuable in complex multisystem diseases and in patients facing communication or social barriers.

## Conclusions

Hereditary ATTR-CM may mimic hypertrophic cardiomyopathy. Multimodality imaging, particularly the integration of CMR and bone scintigraphy, is essential for accurate diagnosis. Recognition of systemic red flags and multidisciplinary evaluation are crucial to enable early disease-modifying therapy.

**Take-Home Messages**

• Current cardiomyopathy guidelines emphasize the importance of recognizing systemic red flags in patients presenting with hypertrophic phenotypes, particularly when conduction abnormalities, neuropathy, or suggestive family history are present.
• In such cases, multimodality imaging integrating cardiac magnetic resonance tissue characterization and bone scintigraphy is critical to establish the diagnosis of transthyretin amyloidosis and allow timely initiation of disease-modifying therapy.
• Multidisciplinary care is essential for diagnosis and management of complex cardiomyopathy.

## Funding Support and Author Disclosures

The authors have reported that they have no relationships relevant to the contents of this paper to disclose.
